# Global research trends in the epidemiology of allergic disorders: a bibliometric and evidence-mapping review

**DOI:** 10.3389/fmed.2026.1874328

**Published:** 2026-06-04

**Authors:** Tina Alhassan, Farida Almarzooqi

**Affiliations:** 1 Department of Internal Medicine, College of Medicine and Health Sciences, United Arab Emirates University, Al Ain, United Arab Emirates; 2 Department of Internal Medicine, Consultant Allergy and Clinical Immunology, Tawam Hospital and Sheikh Tahnoon Bin Mohammed Medical City (STMC), Al Ain, United Arab Emirates

**Keywords:** allergic diseases, asthma, bibliometric analysis, burden of disease, epidemiology, evidence mapping

## Abstract

**Introduction:**

Allergic diseases are highly prevalent worldwide and contribute substantially to morbidity and health-system burden. Bibliometric and evidence-mapping approaches can provide a comprehensive overview of research productivity, collaboration patterns, thematic structure, and distribution of evidence across epidemiologic domains, thereby identifying critical knowledge gaps. This study aimed to characterize global research trends in the epidemiology of allergic disorders from 2016 to 2025 using bibliometric and science-mapping techniques, complemented by evidence mapping.

**Methods:**

We analyzed 1,543 publications indexed in the Web of Science Core Collection and Scopus using Bibliometrix/biblioshiny and VOSviewer to evaluate publication growth, leading journals, authors, countries, collaboration networks, and thematic evolution based on keyword co-occurrence and mapping. An evidence-mapping framework was applied to quantify the distribution of research allergic phenotypes and epidemiologic fields including prevalence, incidence, burden of disease, risk factors, and outcomes.

**Results:**

Scientific production increased steadily (annual growth rate 7.59%), with clear acceleration after 2020, reflecting growing research prioritization of allergic diseases. Scientific production was concentrated within a small group of leading countries, most notably the United States and China, whereas international collaboration remained largely confined to established regional networks rather than broadly distributed global partnerships. Keyword and thematic analyses demonstrated an asthma-centered research structure, with strong emphasis on pediatric populations, prevalence, and risk-factor studies. Evidence mapping revealed a markedly uneven distribution of the literature: risk-factor research dominated across all phenotypes (83–89%), whereas burden-of-disease studies were consistently scarce (<7% across all conditions). Asthma accounted for the largest evidence base across all domains, while non-asthma conditions, including food allergy, anaphylaxis, urticaria/angioedema, and eosinophilic esophagitis, showed comparatively limited and fragmented coverage, particularly for incidence, burden, and patient-centered outcomes.

**Conclusion:**

Global allergy epidemiology research has expanded substantially over the past decade but remains geographically concentrated and structurally imbalanced. The field is heavily centered on asthma and risk-factor research, while critical gaps persist in burden estimation, incidence data, and outcome-focused studies for several allergic phenotypes. Addressing these disparities through broader geographic inclusion and more balanced epidemiologic investigation will be essential to improve the completeness, comparability, and policy relevance of global allergy research.

## 1 Introduction

Allergic disorders comprise a spectrum of immune-mediated conditions including asthma, allergic rhinitis, atopic dermatitis (AD), food allergies, urticaria/angioedema, anaphylaxis, and eosinophilic esophagitis (EoE). These diseases frequently follow the concept of atopic march, in which early-life manifestations such as atopic dermatitis and food allergies may precede the later development of allergic rhinitis and asthma, reflecting shared immunologic mechanisms and disease pathways (1).

Allergic diseases are highly prevalent worldwide and represent a major public health concern. Estimates from the World Allergy Organization indicate that approximately 20–30% of the global population is affected by at least one allergic condition, highlighting their substantial contribution to the global disease burden (2). Epidemiological research on allergic diseases has expanded considerably through population-based surveys, cohort studies, and global burden-of-disease assessments (3). Nevertheless, prevalence patterns vary across regions due to differences in environmental exposures, urbanization, socioeconomic conditions, healthcare access, and diagnostic practices (2, 4). Among allergic diseases, asthma remains one of the most common chronic respiratory conditions and a major contributor to preventable morbidity and mortality worldwide (4, 5). Allergic rhinitis frequently coexists with asthma, reflecting the concept of united airway disease in which inflammation affects both upper and lower airways (6). Other allergic disorders; Atopic dermatitis is highly prevalent worldwide and represents a major contributor to non-fatal disease burden (7). Additionally, up to 40% of children with atopic dermatitis develop food allergies (8, 9).

Beyond their high prevalence, allergic disorders impose a considerable lifelong burden on individuals and healthcare systems. These conditions are associated with sleep disturbance, impaired daily functioning, reduced academic and work productivity, and diminished health-related quality of life. The coexistence of multiple allergic conditions, often described as allergic multimorbidity, further increases clinical complexity and healthcare utilization (2).

Despite the growing volume of research on allergic diseases, the epidemiological literature remains fragmented across disease phenotypes, populations, and geographic regions. Studies are distributed across diverse clinical and public health journals, making it difficult to obtain a comprehensive overview of the global research landscape (10, 11). A recent bibliometric review by Li et al. mapped chronic-care research for comorbid allergic diseases from 1999 to 2024 and identified increasing publication output, leading contributions from the United States, United Kingdom, and China, and hotspots around management strategies, prevalence, risk factors, and quality of life (12). Although that review demonstrates the value of bibliometric approaches for clarifying contributors and research gaps in allergic-disease care, it addressed chronic-care management rather than the epidemiologic evidence base required for burden estimation, surveillance, guideline development, and allergy service planning. In parallel, persistent gaps in disease recognition and reporting remain significant barriers, particularly in low- and middle-income countries, where prevalence estimates may be shaped by underdiagnosis, limited specialist access, and restricted diagnostic capacity (13, 14).

Bibliometric analysis complements traditional reviews by quantitatively evaluating scientific publications. It analyzes metadata such as authorship, affiliations, keywords, and citation networks to assess research productivity, collaboration, and thematic trends within a field (15, 16). Therefore, we conducted a multi-database bibliometric and evidence-mapping review to characterize global research trends in the epidemiology of allergic disorders. Specifically, we analyzed publications from 2016 to 2025 to (i) quantify publication growth and citation impact; (ii) identify the most influential journals, authors, institutions, and countries; (iii) map evolving research themes related to disease burden, prevalence, risk factors, and outcomes; and (iv) classify the evidence base across allergic phenotypes and epidemiologic domains. This overview highlights current strengths and knowledge gaps and may help guide future epidemiologic research, surveillance priorities, and allergy service planning.

## 2 Materials and methods

### 2.1 Study design

This study employed a systematic bibliometric analysis and evidence-mapping approach to assess the global research landscape of population epidemiology research on allergic disorders. Bibliometric indicators were used to evaluate publication output, citation impact, leading contributors, collaboration patterns, and thematic trends. Evidence mapping was used to classify literature by allergic phenotypes and epidemiologic domains. The search and selection process was guided by PRISMA 2020 where applicable (17).

### 2.2 Data sources and search strategy

Data for this study were retrieved from two major bibliographic databases: the Web of Science Core Collection and Scopus. The Web of Science Core Collection served as the primary data source because it provides standardized bibliographic metadata, cited-reference fields, and citation indicators suitable for bibliometric and science-mapping analysis. Scopus was also searched using comparable search strategies and eligibility criteria to enhance coverage of biomedical journals and reduce database-specific indexing bias.

The search covered studies published between 01 January 2016 and 31 December 2025, and data were retrieved on January 03, 2026. The query was structured around four concept blocks: epidemiology and disease burden, allergic disorders and phenotypes, disease determinants, and outcomes and health-service indicators. The full database-specific search syntax is provided in (Table 1).

**Table 1:** Dataset search strategy.

Database field	Search criteria
Identified records from WoSCC and Scopus database	1# (TS) for WoSCC and (TITLE-ABS-KEY) in Scopus = “Disease burden” OR “Population prevalence” OR prevalence OR “Population incidence” OR incidence OR “Cross-sectional study” OR “Cross sectional study” OR “cross-sectional” OR “Cohort study” OR cohort OR “Longitudinal study” OR longitudinal OR “Prospective study” OR prospective OR “Retrospective study” OR retrospective OR “Registry-based study” OR “Registry based study” OR registry OR “Surveillance study” OR surveillance OR “National survey” OR “Community-based study” OR “Community based study” 2# for WoSCC and (TITLE-ABS-KEY) in Scopus = atopy OR “atopic disorder*” OR “atopic disease*” OR “atopic march” OR “allergic disease*” OR “allergic disorder*” OR “allergic phenotype” OR “atopic phenotype” OR “ige-mediated” OR “type i hypersensitiv*” OR “atopic dermatitis” OR eczema OR “allergic rhinitis” OR “hay fever” OR “allergic asthma” OR “bronchial asthma” OR asthma OR “food allergy” OR “ige-mediated food allergy” OR anaphylaxis OR urticaria OR angioedema OR “allergic conjunctivitis” OR “eosinophilic esophagitis” OR “eosinophilic gastrointestinal disorder*” OR EGID* OR EoE OR “type 2 immune disease” OR “allergic multimorbid*” OR “atopy-related disorder*” 3# for WoSCC and (TITLE-ABS-KEY) in Scopus = “Risk factors” OR “Risk factor*” OR “Environmental exposure” OR “Environmental exposure*” OR “Genetic susceptibility” OR “Familial aggregation” OR Consanguinity OR “Socioeconomic factors” OR socioeconomic* 4# for WoSCC and (TITLE-ABS-KEY) in Scopus = Underdiagnosis OR underdiagnos* OR Misdiagnosis OR misdiagnos* OR “Health care utilization” OR “Healthcare utilization” OR Hospitalization OR hospitalisation* OR “Hospitalization rates” OR “Hospitalisation rates” OR Mortality OR Morbidity OR “Quality of life” OR “quality of life”
Time span (index date): 2016–2025	# 5 = #1 AND #2 AND #3 AND #4

Records were identified through searches in the Web of Science Core Collection (WoSCC) and Scopus databases (2016–2025). The search combined terms related to epidemiological study designs and disease burden (#1), atopic and allergic diseases (#2), risk factors (#3), and diagnostic or health outcomes (#4). The final search set was created using #1 AND #2 AND #3 AND #4.

### 2.3 Inclusion and exclusion criteria

English-language original articles addressing the population epidemiology of allergic disorders were eligible for inclusion. Eligible publications included studies reporting or analyzing prevalence, incidence, disease burden, risk factors, determinants, outcomes, quality of life, healthcare utilization, morbidity, or mortality related to allergic phenotypes.

**Figure 1:**
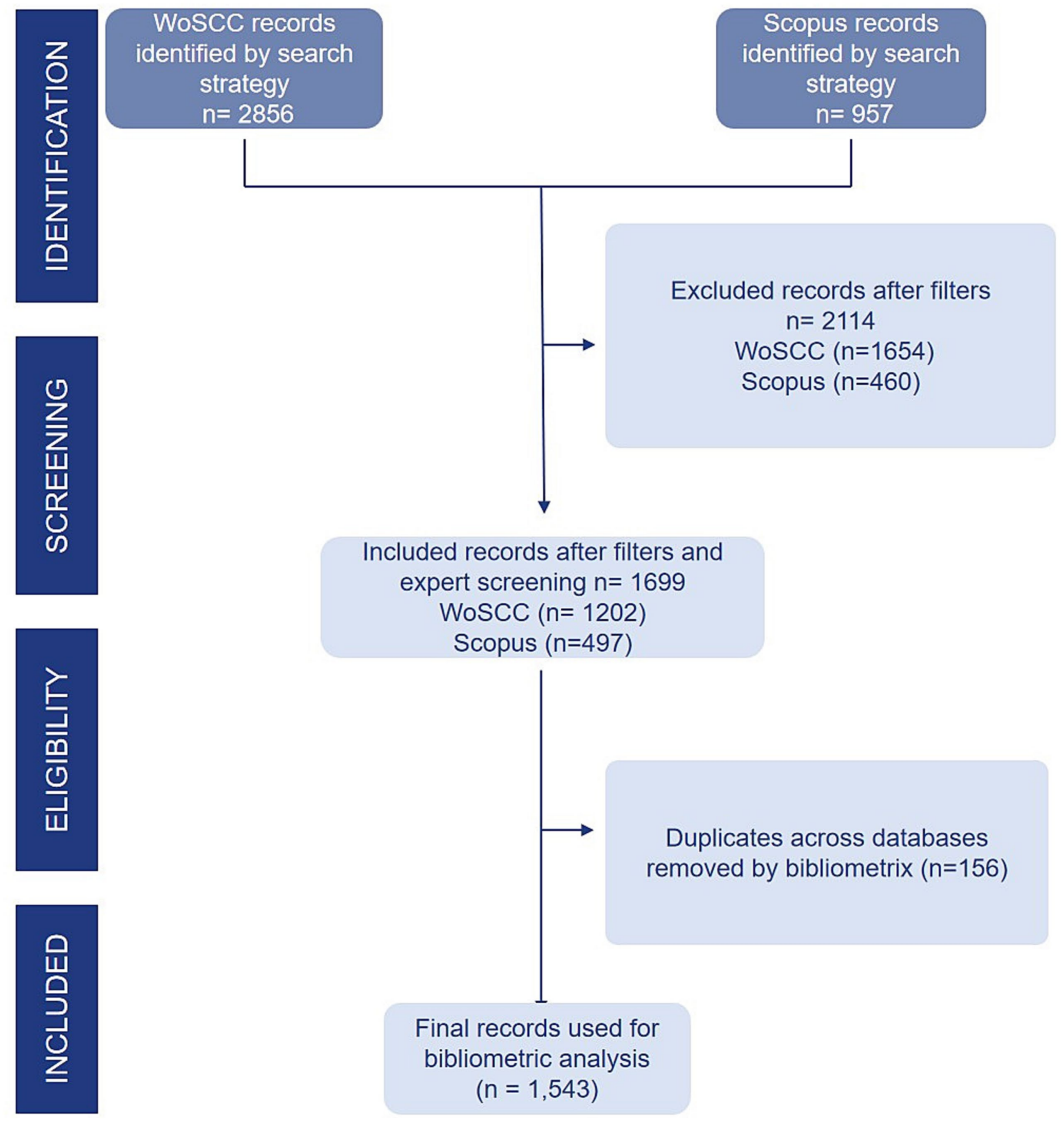
PRISMA flow diagram of study selection. The study selection process for publications between 2016 and 2025 is illustrated using a PRISMA-style flow diagram. A total of 2,856 records were identified from the Web of Science Core Collection (WoSCC) and 957 from Scopus. After applying filters for publication year, language, and document type, followed by relevance screening, 1,202 WoSCC records and 497 Scopus records met the eligibility criteria. The datasets were subsequently merged, and cross-database duplicates (*n* = 156) were removed. A total of 1,543 unique publications were included in the final bibliometric analysis.

Reviews, early-access items, meeting abstracts, editorials, letters, book chapters, conference proceedings, meeting records, and retracted papers were excluded. The study selection process was summarized using a PRISMA-style flow diagram to enhance transparency (Figure 1) (17). The search strategy retrieved 2,856 records from the Web of Science Core Collection and 957 records from Scopus. After applying language and document-type restrictions, titles, abstracts, and author keywords were screened for relevance to the population epidemiology of allergic disorders. A total of 1,202 Web of Science Core Collection articles and 497 Scopus articles met the eligibility criteria and were included before deduplication.

### 2.4 Data synthesis and analysis

The 1,699 eligible records were imported into R software, version 4.5.2, and analyzed using the Bibliometrix/Biblioshiny package (15, 16). Cross-database deduplication was performed using DOI matching, followed by exact title matching when DOI information was unavailable. A total of 156 overlapping records were removed, resulting in 1,543 unique articles included in the final bibliometric analysis (Figure 1).

Full bibliographic metadata were extracted for each record, including authors, title, publication year, source journal, affiliations and countries, keywords, citation data, and, where available, referenced bibliographies. Data cleaning and harmonization were performed by standardizing country names, institution names, journal titles, and keywords. Variant and similar terms were manually reviewed and merged where appropriate to improve consistency in keyword and thematic analysis.

Descriptive bibliometric indicators were used to assess annual publication trends, source productivity, author productivity, country productivity, international collaboration, and citation impact. Annual publication counts and citation indicators were exported from Biblioshiny and combined in Microsoft Excel for visualization. Trend topics and thematic maps were generated using Bibliometrix through the Biblioshiny interface (15, 16). Collaboration networks and keyword co-occurrence maps were constructed using VOSviewer (18).

### 2.5 Evidence-mapping analysis

To extend the bibliometric analysis, the final deduplicated dataset was organized into a phenotype-by-domain matrix using published evidence-mapping approaches (19, 20). Publications were classified by allergic phenotype (asthma, allergic rhinitis, atopic dermatitis, food allergies, urticaria/angioedema, anaphylaxis, and eosinophilic esophagitis) and by epidemiologic domain (prevalence, incidence, global burden of disease [GBD] burden, risk factors, and outcomes/quality of life).

Classification was based on review of titles, abstracts, and author keywords. Prevalence and incidence were assigned to studies estimating disease frequency or new occurrence; GBD burden was assigned to publications reporting standardized burden metrics such as disability-adjusted life-years, years lived with disability, or GBD-derived estimates; risk factors included environmental, genetic, socioeconomic, demographic, and clinical determinants; and outcomes/quality of life included patient-centered outcomes, health-related quality of life, healthcare utilization, morbidity, and mortality.

Because individual publications could address more than one phenotype or epidemiologic domain, records were assigned to all applicable categories. The analysis was performed in R (version 4.5.2) using the final dataset of 1,543 publications. For each phenotype-domain combination, absolute counts and within-phenotype percentages were calculated, and the results were visualized as a heatmap to identify areas of relative evidence concentration and evidence gaps across allergic phenotypes and epidemiologic domains.

## 3 Results

### 3.1 Bibliometric overview

**Figure 2:**
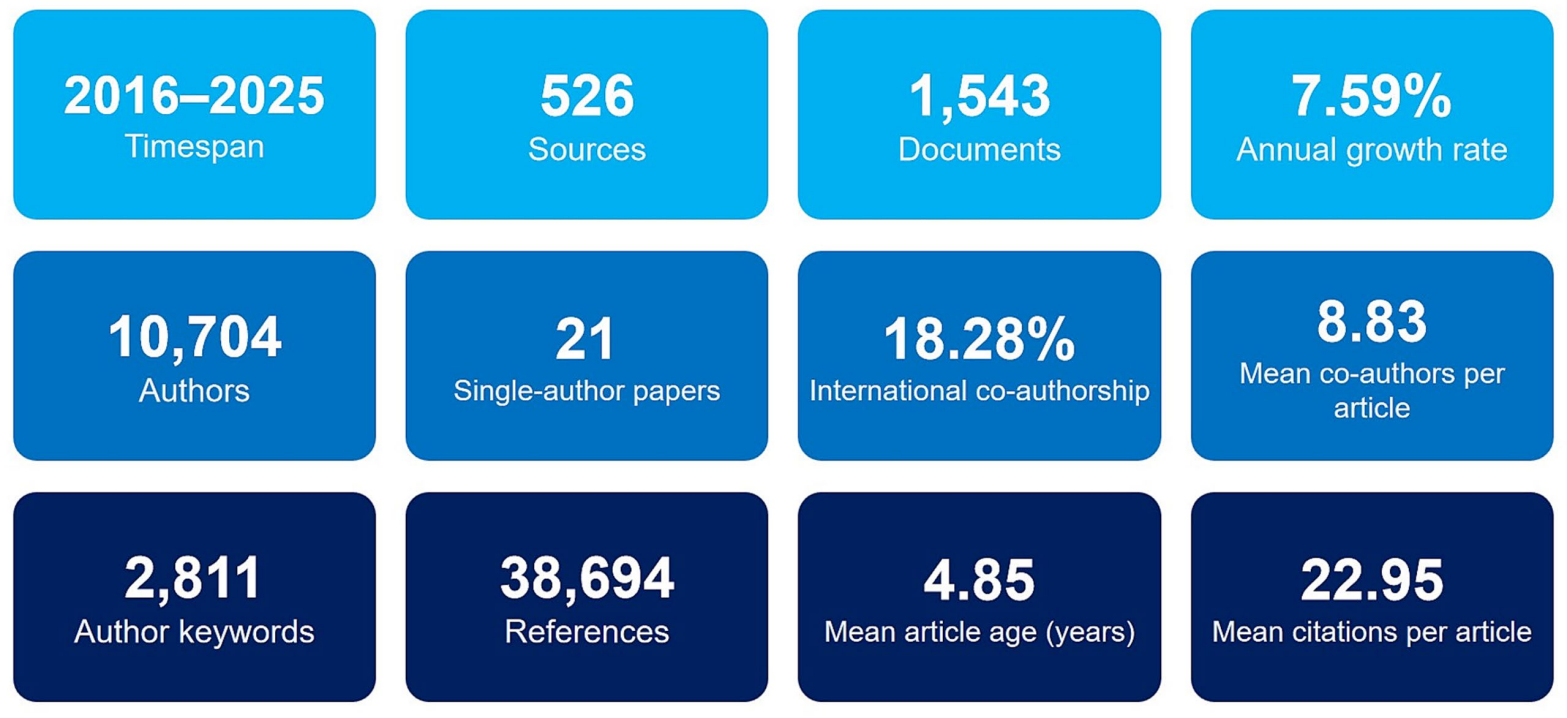
Descriptive bibliometric summary. Summary of bibliometric indicators for the analyzed publications (2016–2025), including numbers of documents, sources, authors, and keywords; authorship pattern; mean co-authors per document; international co-authorship rate; annual growth rate; mean document age; and average citations per document, derived from the bibliometric dataset.

The final dataset comprised 1,543 unique publications on the epidemiology of allergic disorders published between 2016 and 2025. These articles were distributed across 526 sources and reflected a large multi-author research community, with only 21 single-authored papers, underscoring the strongly collaborative nature of the field. The mean number of co-authors per document was 8.83, whereas 18.28% of publications involved international co-authorship, indicating that large research teams were common but cross-border collaboration remained comparatively limited. Overall field performance was robust, with an annual growth rate of 7.59%, a mean age of 4.85 years, and an average of 22.95 citations per document (Figure 2). Collectively, these indicators describe a rapidly expanding, citation-active, and highly networked research field.

### 3.2 Trends in scientific production and citation impact

**Figure 3:**
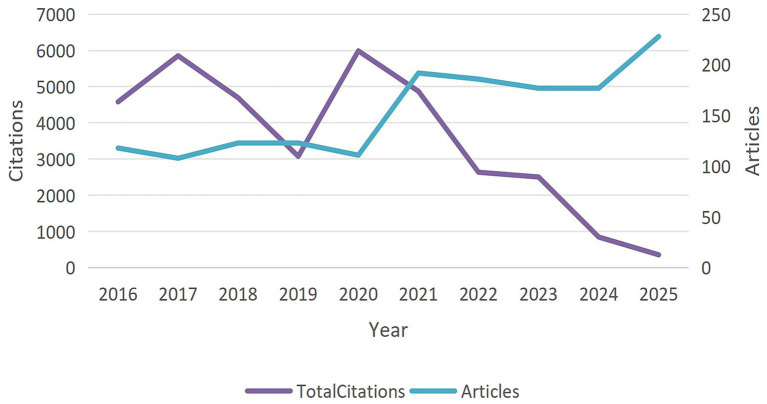
Annual scientific production and citations. Yearly number of publications and total citations in the analyzed allergy epidemiology dataset from 2016 to 2025. The purple line represents annual publication output, showing a steady increase over time with acceleration after 2020. The blue line represents total citations per publication year, which peaked around 2017 and 2020 before declining in more recent years, likely reflecting the shorter citation window available.

Scientific output increased steadily across the study period, rising from approximately 120 publications in 2016 to more than 230 in 2025, with a visibly steeper upward trajectory after 2020 (Figure 3). This pattern indicates that allergy epidemiology has gained momentum as a research priority in recent years. Citation counts followed a different trajectory, peaking around 2017 and 2020 and declining thereafter. Rather than suggesting reduced scholarly influence, this decline is more plausibly explained by the shorter citation window available for recently published papers. Taken together, the production and citation curves suggest a field that is expanding in volume while continuing to generate influential scholarship.

### 3.3 Source concentration and author productivity

Although the literature was distributed across many journals, publication activity was concentrated in a core group of specialty sources. The most productive journal was Journal of Allergy and Clinical Immunology: In practice (77 documents), followed by Journal of Asthma (72), PLOS ONE (35), and Journal of Allergy and Clinical Immunology (32) (Table 2). This distribution indicates that allergy epidemiology is anchored primarily in established allergy, respiratory, and general biomedical journals, with a relatively small number of outlets serving as key dissemination hubs for the field. At the author level, Janson C and Kim J were the most prolific contributors, with 15 publications each, followed by Kim SH and Phipatanakul W with 13 publications each. When co-authorship was adjusted using fractionalized counting, Lee S showed the highest fractional contribution (2.42), followed by Kim J (1.91) and Janson C (1.76), suggesting sustained individual contribution beyond participation in large collaborative terms. Overall, the authorship pattern indicates that the field is driven by recognizable core of recurrent contributors working within a broader multi-author research environment (Table 2).

**Table 2:** Journals and author production.

Rank	Journal	Journal articles (*n*)	Author	Author articles (*n*)	Author articles (fractional)
*1*	Journal of Allergy and Clinical Immunology-in Practice	77	Janson C	15	1.76
*2*	Journal of Asthma	72	Kim J	15	1.91
*3*	PLoS One	35	Kim SH	13	1.54
*4*	Journal of Allergy and Clinical Immunology	32	Phipatanakul W	13	1.56
*5*	Respiratory Medicine	31	Li J	12	1.44
*6*	Pediatric Allergy and Immunology	29	To T	12	1.6
*7*	Journal of Asthma and Allergy	28	Hasegawa K	11	1.41
*8*	Pediatric Pulmonology	27	Zhang J	11	1.29
*9*	World Allergy Organization Journal	27	Lee S	10	2.42
*10*	BMJ Open	23	Matsui EC	10	1.66

This table presents the top 10 most productive journals ranked by the number of documents published in the dataset, as well as the top 10 most productive authors ranked by the number of documents, including fractionalized publication counts, where credit is proportionally allocated among co-authors to account for multi-authored papers.

### 3.4 Geographic distribution and international collaboration

**Figure 4:**
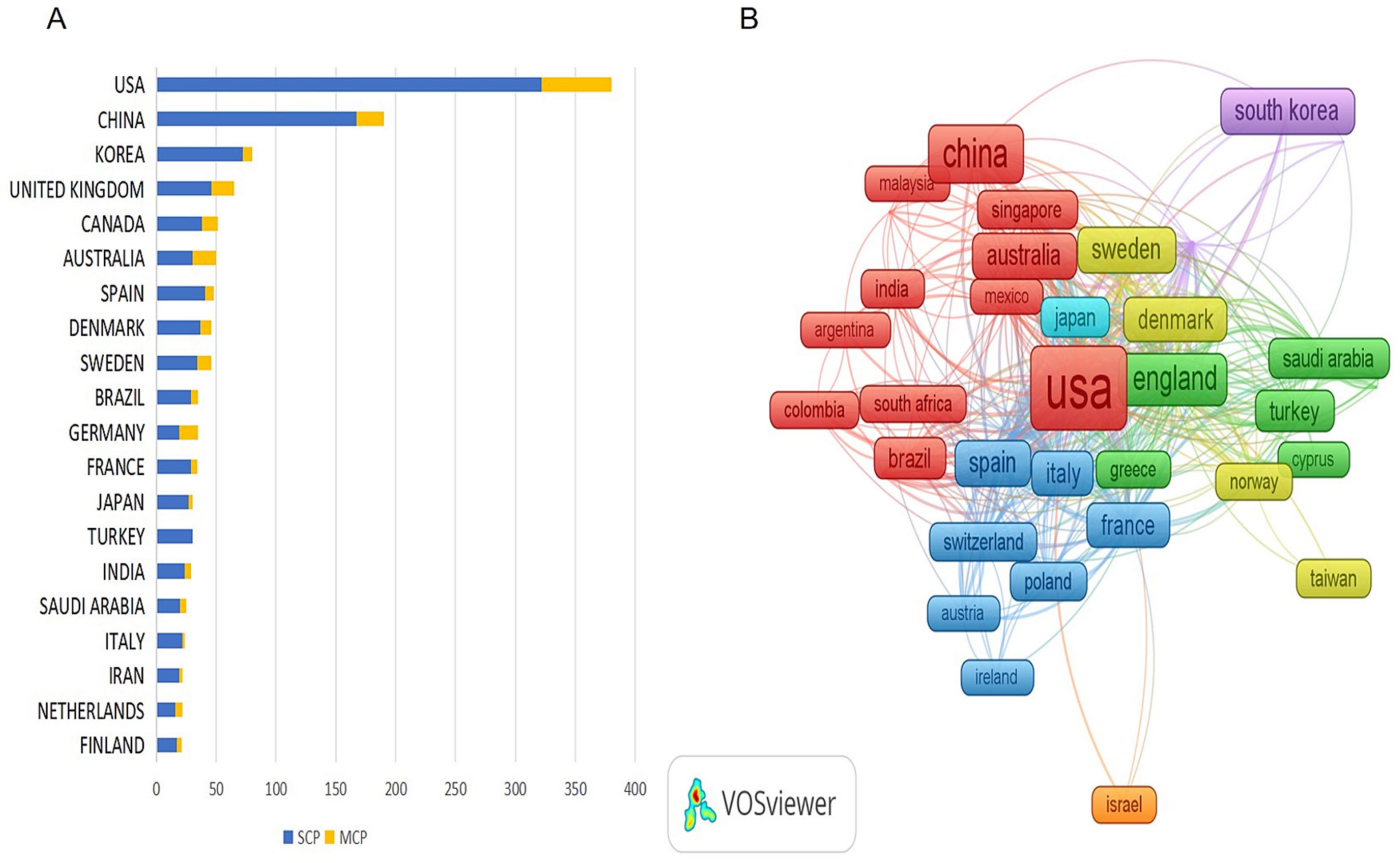
Country productivity and international collaboration network. **(A)** Distribution of corresponding author countries categorized into single-country publications (SCP) and multi-country publications (MCP), indicating the extent of domestic versus international research output. **(B)** Country-level collaboration network visualized using VOSviewer, where each node represents a country and node size reflects publication output; links indicate co-authorship relationships, and link thickness represents collaboration strength (total link strength). Colors denote collaboration clusters of countries that frequently collaborate with each other.

Research productivity was concentrated in a limited number of countries. Based on corresponding-author affiliation the United States and China produced the highest volume of publications, with output dominated by single-country publications, whereas several European countries showed a relatively greater share of multi-country publications (Figure 4A). The international collaboration network further demonstrated that the United States occupied the most central bridging position, linking multiple global clusters. China clustered predominantly with Asia-Pacific collaborators, while European countries formed dense intra-regional networks (Figure 4B). These findings indicate that global allergy epidemiology is characterized by strong regional collaboration structures rather than evenly distributed worldwide integration. It should be noted that prominence in the collaboration network does not necessarily mirror corresponding-author publication rank. The productivity panel reflects corresponding-author output, whereas the network visualization reflects co-authorship connectivity and link strength. Accordingly, some countries with more modest publication totals may still appear prominently in the network because they participate more frequently in multinational collaborations, while some higher-output countries may be less visible if a larger share of their publications are produced through domestic rather than international partnerships. This may also reflect varying degrees of internationalisation across countries, with lower network visibility potentially indicating more limited engagement in international collaborative research. They also show that knowledge production remains concentrated in a small number of high-output settings, with comparatively limited visibility from many other regions.

### 3.5 Keyword analysis and conceptual structure

Author keywords could be grouped into four broad thematic categories: disease phenotype (asthma, allergic rhinitis, and atopic dermatitis), population/specialty descriptors (pediatric), epidemiologic concepts (prevalence and risk factors), and patient-centered outcomes (quality of life). Within this structure, asthma remained the dominant disease-related term, indicating that the field is centered on common allergic phenotypes while also emphasizing pediatric populations, population-based epidemiologic questions, and quality-of-life outcomes.

**Figure 5:**
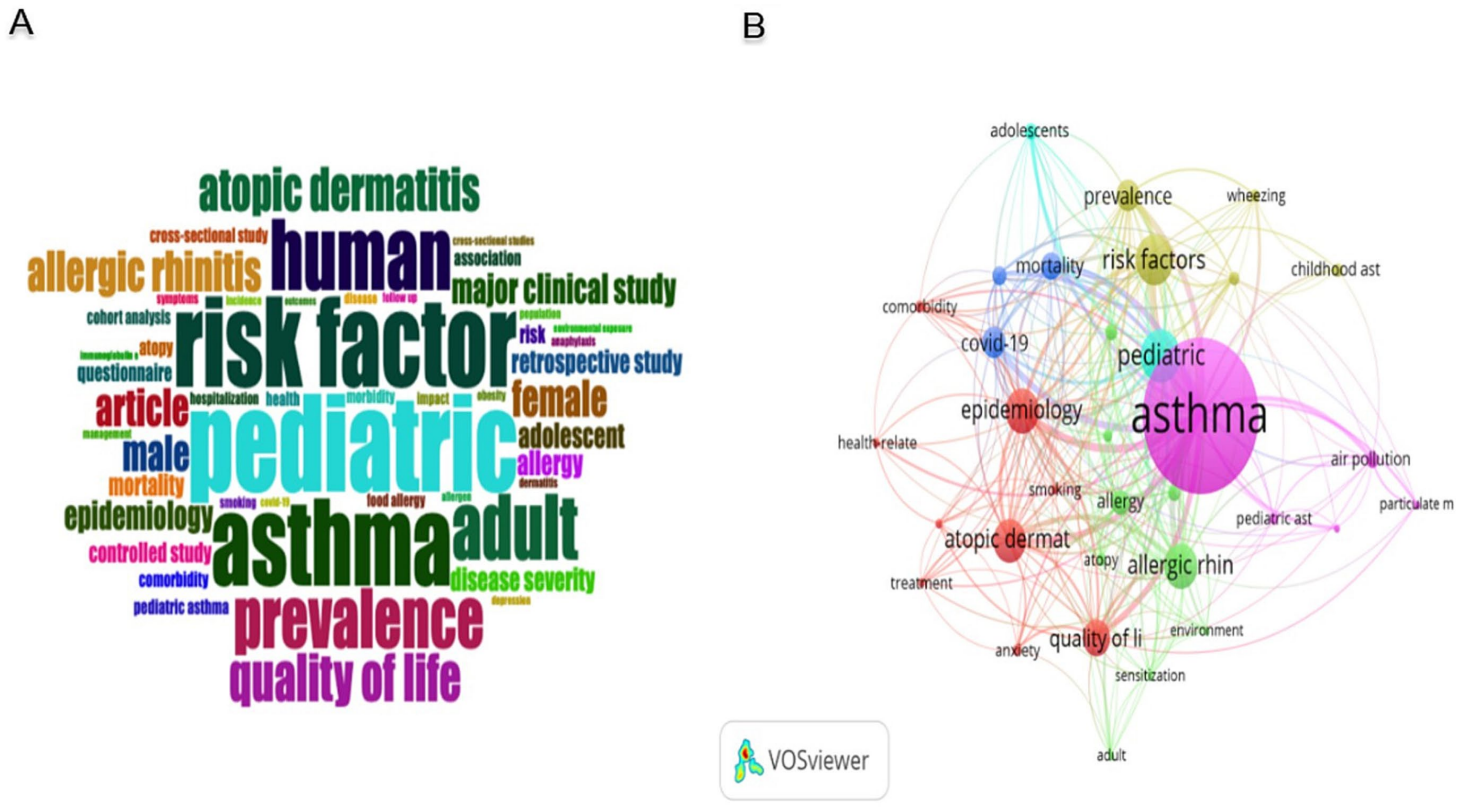
Author keyword frequency and co-occurrence structure. **(A)** Word cloud of author keywords in which word size reflects the frequency of occurrence in the dataset, highlighting the most prominent research topics; colors are used for visual differentiation only. **(B)** Keyword co-occurrence network generated in VOSviewer, where nodes represent keywords and node size corresponds to keyword frequency; links indicate co-occurrence within the same documents, and link thickness reflects co-occurrence strength (total link strength). Colors represent keyword clusters identified by VOSviewer.

In the word cloud, asthma-related and outcome-oriented terms were the most visually prominent (Figure 5A). In the co-occurrence network, asthma formed the largest and most central node and was strongly connected to pediatric research, risk-factor studies, and environmental exposures such as air pollution (Figure 5B). Additional clusters around atopic dermatitis, allergic rhinitis, epidemiology, and quality of life outcomes indicate that the literature is organized into recognizable subdomains, but these remain connected to an asthma –centered core. Overall, the keyword structure shows that allergy epidemiology is conceptually dominated by allergic phenotypes and patient-centered outcomes.

### 3.6 Thematic structure of allergy epidemiology research

**Figure 6:**
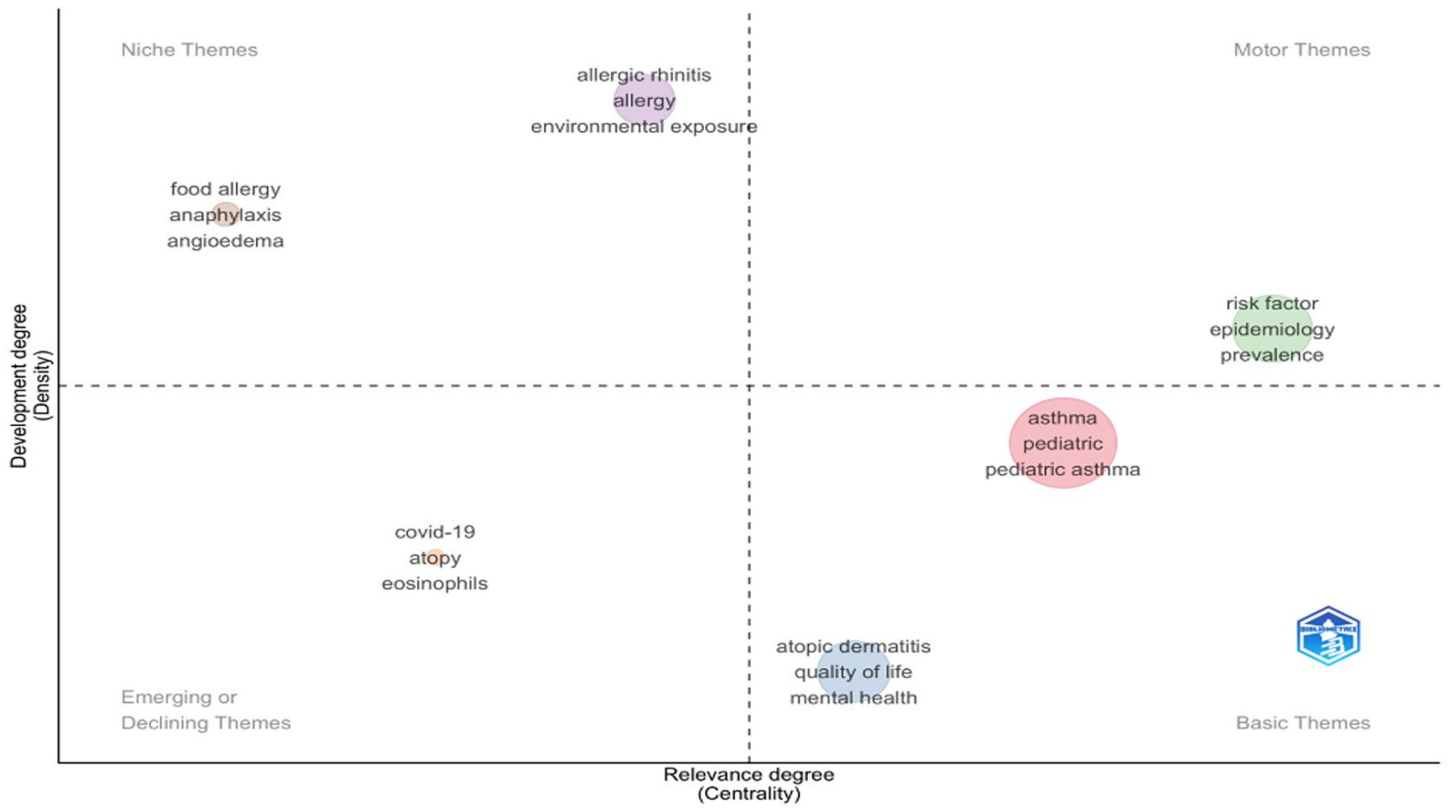
Keyword thematic map. Thematic map derived from keyword clustering, plotted by centrality (*x*-axis; relevance and connectedness to other themes) and density (*y*-axis; internal development or maturity). Themes in the upper-right quadrant represent motor themes; lower-right basic themes; upper-left niche themes; and lower-left emerging or declining themes. Bubble size reflects the relative weight of each theme based on the frequency of associated keywords.

The thematic map identified four major domains according to centrality and density (Figure 6). Motor themes were dominated by risk factors, epidemiology, and prevalence. Demonstrating that population burden and disease determinants constitute the most developed and influential area of the field. Basic themes included asthma, pediatric asthma, and pediatric research, as well as a cluster linking atopic dermatitis with quality of life and mental health; these topics appear foundational to the field and widely connected, although some remain conceptually less mature. Niche themes, including food allergies, anaphylaxis, angioedema, allergic rhinitis and environmental exposure. In this context, placement in the niche quadrant indicates a relatively specialized and less centrally connected keyword structure within the thematic map. Emerging or declining themes included COVID-19, atopy, and eosinophils, suggesting topics that are either newly developing or losing prominence. Overall. The thematic structure indicates a mature central literature focused on prevalence and risk-factor epidemiology, whereas several clinically important non-asthma phenotypes remain more specialized, fragmented, or peripheral within the field.

### 3.7 Evidence mapping across allergic phenotypes and epidemiologic domains

**Figure 7:**
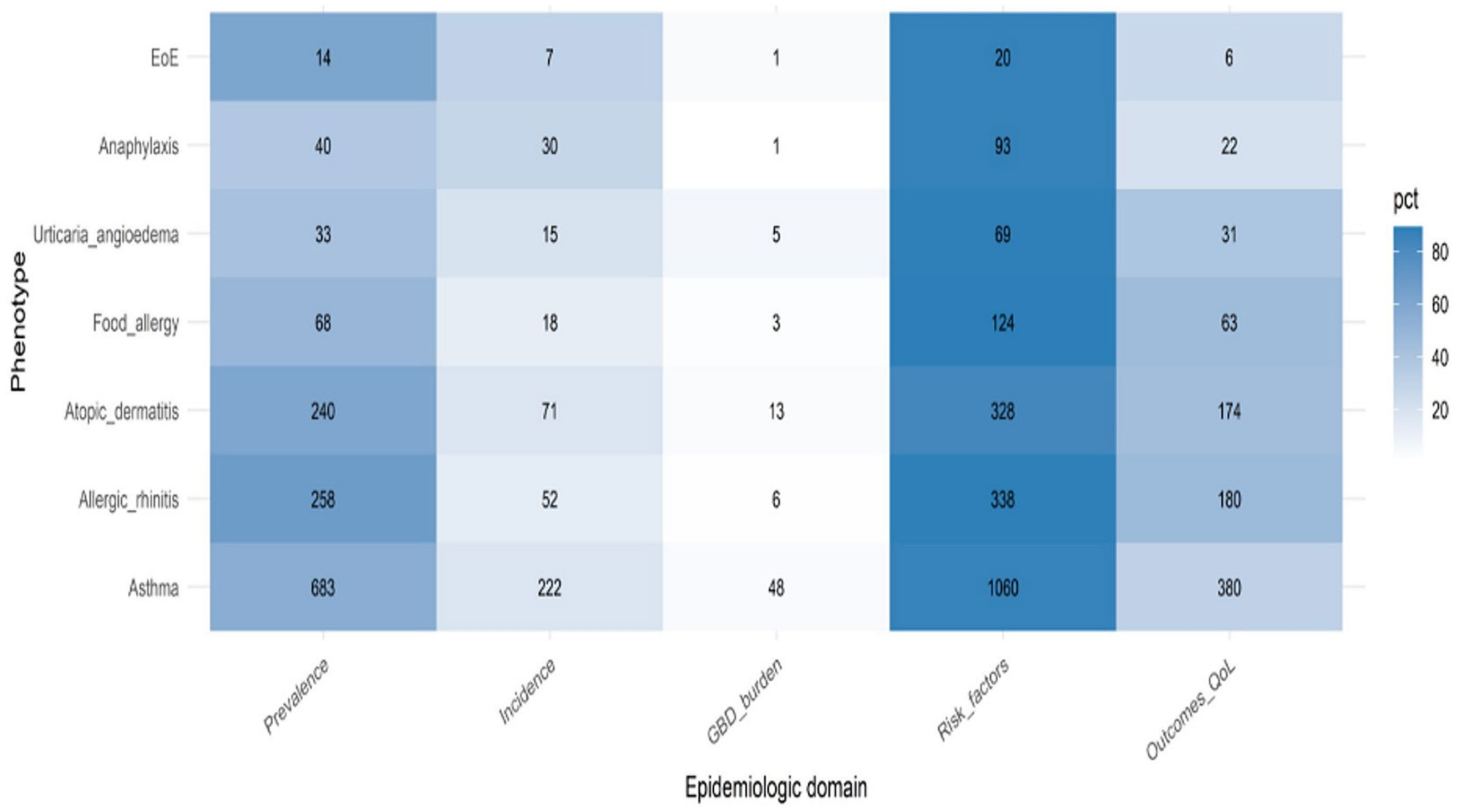
Evidence mapping heatmap across allergic phenotypes and epidemiologic domains. Heatmap showing the distribution of publications across seven allergic phenotypes (asthma, allergic rhinitis, atopic dermatitis, food allergy, urticaria/angioedema, anaphylaxis, and eosinophilic esophagitis) and five epidemiologic domains (prevalence, incidence, global burden of disease (GBD burden), risk factors, and outcomes/quality of life). Cell labels indicate the absolute number of publications, whereas color intensity represents the percentage of publications within each phenotype addressing that domain. Darker shading indicates a greater concentration of evidence within a phenotype. Because individual publications could contribute to more than one domain, percentages within a phenotype are not mutually exclusive and do not sum to 100%. The figure highlights the predominance of risk-factor studies across all phenotypes and the relative scarcity of GBD-burden research.

The evidence map summarizes the distribution of allergy epidemiology literature across phenotypes and epidemiologic domains (Figure 7). Across all phenotypes, risk-factor research was the most heavily represented domain. Asthma showed the largest absolute evidence base in every domain, including prevalence (*n* = 683, 56.0%), incidence (*n* = 222, 18.2%), global burden of disease (GBD) (*n* = 48, 3.9%), risk factors (*n* = 1,060, 86.9%), and outcomes/quality of life (*n* = 380, 31.1%), confirming that it dominates not only the conceptual structure of the field but also its empirical evidence base. Allergic rhinitis and atopic dermatitis demonstrated broader epidemiologic coverage than other non-asthma conditions, particularly for prevalence and outcome-related research, with prevalence represented in 67.7% (*n* = 258) and 60.8% (*n* = 240) of publications, respectively, and outcomes/quality of life research in 47.2% (*n* = 180) and 44.1% (*n* = 174). These distributions suggest comparatively more mature literature on population frequency and patient burden for these phenotypes.

By contrast, food allergies, urticaria/angioedema, anaphylaxis, and eosinophilic esophagitis represented smaller and more uneven evidence domains. Although incidence studies were proportionally more visible in anaphylaxis (28.0%, *n* = 30) and eosinophilic esophagitis (30.4%, *n* = 7), the absolute volume of this literature remained modest. The most consistent evidence gap across all phenotypes was GBD-burden research, which accounted for only 0.9% of the anaphylaxis publications (*n* = 1), 1.6% of allergic rhinitis (*n* = 6), 2.2% of food allergies (*n* = 3), 3.3% of atopic dermatitis (*n* = 13), 3.9% of asthma (*n* = 48), 4.3% of eosinophilic esophagitis (*n* = 1), and 6.4% of urticaria/angioedema (*n* = 5). Overall, the heatmap demonstrates a structurally uneven evidence base: the literature is strongly weighted toward identifying risk factors and estimating prevalence, whereas standardized burden assessment and, for several non-asthma conditions, incidence and patient-centered outcomes remain comparatively underdeveloped.

## 4 Discussion

In this bibliometric and evidence-mapping review, global research on the epidemiology of allergic disorders increased steadily over the past decade, with clear acceleration after 2020. Consistent with growing recognition of allergic disorders as major contributors to morbidity, impaired quality of life, health-care utilization (2, 3, 5, 11). This upward trend is likely multifactorial, reflecting a convergence of epidemiological, social, environmental, and health-system drivers. Epidemiologically, it may be linked to increasing recognition of the global burden of allergic disease and to the expansion of standardized multicountry surveillance and burden-estimation initiatives, which have created greater demand for prevalence, risk-factor, and outcome data (2, 3, 5, 21). At the same time, growing attention to socioeconomic disparities, environmental injustice, air pollution, and climate-related allergen exposure may have further broadened the research agenda in allergy epidemiology (22, 23). In addition, the COVID-19 period likely heightened the policy and health-system visibility of respiratory and immune-mediated conditions, thereby further amplifying research interest in asthma and related allergic diseases (11, 24). Despite this expansion, however, the evidence base has not developed evenly. Two key patterns emerged from the analysis are discussed below: (i) concentration of research output and citation impact within a limited number of countries; and (ii) asthma representing the most conceptually central and methodologically developed phenotype, alongside uneven epidemiologic development across other allergic conditions.

### 4.1 Geographic research concentration

Global analysis demonstrate that allergic disease constitute a substantial and geographically heterogeneous public health burden (3, 4). However, our bibliometric findings indicate a marked imbalance in the distribution of research output informing these estimates. Scientific productivity and citation impact remain concentrated in high-income countries, while low- and middle-income countries contribute comparatively fewer publications (14). This disparity raises concerns regarding the representativeness and generalizability of burden estimates and clinical guidance derived predominantly from high-income settings, particularly given established geographic variation in environmental exposures and allergen profiles (25–27).

The Middle East and North Africa (MENA) region reflect this broader pattern of underrepresentaion. Available studies suggest significant sensitization to regionally prevalent aeroallergens; however, the evidence base remains heterogeneous in design, methodology, and surveillance scope (25, 27–29). Limited standardization across studies restricts cross-country comparability and may affect the precision of regional burden estimation (30). Environmental determinants characteristic of arid and semi-arid climates differs from those of temperate regions, which may limit the direct transferability of risk prediction models developed in Europe or North America (25, 26). Genetic and demographic characteristics may further influence disease expression in MENA populations. High consanguinity rates are associated with an increased prevalence of inborn errors of immunity, some of which present with overlapping atopic features (31, 32). Such gene–environmental interactions may modify susceptibility and clinical expression, underscoring the importance of regionally representative epidemiologic data rather than reliance on extrapolated risk frameworks (33, 34). Addressing these gaps will require strengthened regional research infrastructure, improve data harmonization, and more balanced international collaboration networks (14, 35). Established initiatives such as global Burden of Disease program and the Global Asthma Network illustrate the feasibility of standardized cross-country methodologies (5, 21). Expansion of similar coordinated approaches across allergic diseases may enhance representiveness, reduce evidence gaps in under-studied regions, and improve the policy relevance of global allergy epidemiology (5, 14, 21).

### 4.2 Asthma-centered structure and uneven epidemiologic development across other allergic phenotypes

An integrated reading of the keyword analysis, thematic map, and phenotype-domain matrix suggests that the epidemiology literature on allergic disease is unevenly developed across phenotypes, with asthma occupying the most conceptually central and methodologically mature position. Asthma dominated the keyword structure, remained embedded within the core thematic architecture of the field, and was the only phenotype supported by a comparatively broad evidence base spanning prevalence, incidence, risk factors, outcomes, and burden-related research. This pattern likely reflects the availability of long-standing international surveillance and standardization frameworks, particularly the International Study of Asthma and Allergies in Childhood (ISAAC), the Global Asthma Network, and the Global Initiative for Asthma (GINA), which have made asthma more amenable to repeated multicountry assessment than most other allergic disorders (4, 5, 21, 36, 37). Consequently, asthma appears to have shaped not only the visibility of the field, but also the dominant epidemiologic questions through which allergic disease has been studied. This interpretation is also consistent with prior bibliometric work in asthma and allergic rhinitis showing broader international collaboration and more developed hotspot structure in asthma research (38).

Allergic rhinitis showed a distinct pattern. Although it was one of the most prominent phenotype-related topics after asthma, its placement in the niche quadrant does not indicate limited importance. Rather, it reflects thematic specialization, with the literature concentrated around closely related topics such as phenotype/endotype classification, local allergic rhinitis, immunotherapy, care pathways, and patient-centered outcomes including sleep and quality of life, rather than broader epidemiologic themes such as prevalence, risk factors, and disease burden that dominated asthma research (6, 38–42). This interpretation is consistent with the broader rhinitis literature and recent bibliometric analyses highlighting these specialized areas as major research directions in the field (40–42). Therefore, the niche-map position of allergic rhinitis likely reflects conceptual specialization within a substantial body of literature rather than marginal epidemiologic relevance.

By contrast, atopic dermatitis occupied a more foundational position in the thematic structure. Its placement within the basic-theme quadrant suggests stronger conceptual connectedness to the broader field, even though its internal thematic development was less dense than that of the motor themes. This profile is consistent with the role of atopic dermatitis as a major component of allergy epidemiology through its links to prevalence, multimorbidity, patient burden, and quality-of-life research (1, 7, 43). Nevertheless, compared with asthma, its literature remained less consistently developed in broader burden-oriented analyses, indicating that conceptual connectedness has not yet translated into equally mature coverage across all epidemiologic domains (7, 43).

An even more methodologically constrained epidemiologic profile was observed for food allergies and anaphylaxis. In both conditions, literature remains more developed around determinants and clinical characterization than around burden estimation or broader outcomes. This likely reflects persistent difficulties in case ascertainment and standardization, particularly when population studies rely on self-report or sensitization-based proxies and when severe acute events are not consistently captured routine datasets (8, 44, 45). These barriers limit the development of stable, comparable epidemiologic profiles and help explain why these phenotypes remain more specialized within the broader research landscape.

A related limitation is seen in urticaria/angioedema and eosinophilic esophagitis remained comparatively smallest and least balanced evidence domain. For these conditions, the restricted epidemiologic scope likely reflects heterogeneity in diagnostic labeling, reliance on specialist evaluation, and, in the case of eosinophilic esophagitis, the need for endoscopic and histologic confirmation (46–48). These factors make large-scale surveillance more difficult and reduce comparability across studies, which in turn may contribute to their more peripheral position in the thematic structure of the field.

Taken together, these findings indicate that the current allergy epidemiology literature is stronger at identifying determinants of disease than at quantifying comparative burden across phenotypes. This imbalance is methodologically understandable: cross-sectional and cohort designs are well suited to studying prevalence, incidence, and risk-factor associations, whereas burden modeling depends on reference case definitions, standardized coding systems, adjustment across alternative measurement methods, and sufficiently comparable multicountry data inputs (49–51). Unlike prior bibliometric analysis focused on allergic skin disorders, asthma versus-rhinitis research, or chronic-care management of comorbid allergic diseases (10, 12, 38), our review shows that the critical gap lies not only in which phenotypes receive attention, but also in which kinds of epidemiologic evidence are available for each phenotype.

### 4.3 Future directions

The imbalances identified in this analysis highlight several priorities for future research. Greater standardization of diagnostic criteria and core outcome measures, particularly for food allergy, chronic spontaneous urticaria, and eosinophilic esophagitis, would reduce heterogeneity in prevalence estimates and strengthen global evidence synthesis (8, 46, 48). Integrating allergic multimorbidity into epidemiologic platforms may also improve understanding of disease trajectories across the atopic spectrum and reduce fragmentation across allergic phenotypes (1). The evidence-map findings further support investment in burden-of-disease modeling and outcomes/quality-of-life for underrepresented phenotypes, alongside stronger international partnerships and expanded research capacity in underrepresented regions to support more representative multicountry cohorts (3, 14).

### 4.4 Strengths and limitations

This review has several strengths, including the use of two major bibliographic databases, cross-database deduplication, combined bibliometric and evidence-mapping methods, and a phenotype-by-domain matrix designed to make gaps in the epidemiologic evidence base visible. Nevertheless, several limitations should considered. First, the analysis was restricted to the Web of Science Core Collection and Scopus. These databases were selected because they provide standardized bibliographic metadata and cited-reference fields suitable for citation-based bibliometric mapping, while their combined use broadens journal coverage relative to a single-database design. However, reliance on these sources may still underrepresent regional or less visible journals (52, 53). OpenAlex has emerged as a comprehensive open bibliographic database with broad journal coverage and improved geographical representation compared with traditional indexing systems (52, 54). Nevertheless, recent evaluations have also identified limitations in metadata consistency, citation standardization, and infrastructure maturity that may affect reproducibility in citation-based bibliometric analyses (54, 55). Therefore, OpenAlex was not incorporated into the present study. Future bibliometric updates could integrate OpenAlex alongside WoSCC and Scopus to further evaluate the robustness of the observed geographic and thematic patterns (52, 54, 55). Second, restricting inclusion to English-language publications may have introduced language bias (14, 53, 56–60). Although the prominence of the United States and China likely reflects substantial research productivity, the visibility of the United States may be further amplified by English being its primary language, whereas China increasingly publishes in English-language indexed journals to support international scientific dissemination. Consequently, studies from settings where local-language publication remains common may be underrepresented. This should be considered when interpreting the geographic distribution of publications and international research influence identified in the present analysis (14, 53, 56–60). Third, because the study was a bibliometric and evidence-mapping review across allergic phenotypes and epidemiologic domains, the findings rely on author-provided titles, abstracts, and keywords; variability and inconsistency in terminology may have resulted in omission or misclassification of pertinent studies. Finally, recent publications had shorter citation windows, which may have affected citation-based indicators.

## 5 Conclusion

Global research on the epidemiology of allergic disorders expanded steadily between 2016 and 2025, reflecting growing recognition of their clinical and public-health importance, However the evidence base remains geographically concentrated, with limited representation from many lo- and middle- income regions. This imbalance may restrict the generalizability of current estimates, particularly in settings with distinct environmental exposures, genetic backgrounds, healthcare access, and diagnostic capacity.

The field also remains structurally centered on asthma, which has the most mature and comprehensive epidemiologic evidence base. In contrast, the other allergic conditions, including food allergies, anaphylaxis, urticaria/angioedema, and eosinophilic esophagitis, show narrower and less balanced coverage. Across phenotypes, the literature is dominated by prevalence and risk factor research, whereas burden-of-disease estimation, incidence data and patient-centered outcomes remain comparatively underdeveloped.

Future allergy epidemiology research should prioritize broader geographic inclusion, standardized phenotype-specific methods, coordinated surveillance beyond asthma, and greater attention to burden and outcome measures. These steps are essential to build a more balanced, comparable, and globally representative evidence base for allergic diseases.

## Data Availability

The data analyzed in this study is subject to the following licenses/restrictions: Raw records from Web of Science Core Collection and Scopus cannot be publicly shared due to database licensing restrictions. Derived data are available from the corresponding author upon reasonable request. Requests to access these datasets should be directed to FA, famarzooqi@uaeu.ac.ae.
